# Segmentation-Based Classification Deep Learning Model Embedded with Explainable AI for COVID-19 Detection in Chest X-ray Scans

**DOI:** 10.3390/diagnostics12092132

**Published:** 2022-09-02

**Authors:** Nillmani, Neeraj Sharma, Luca Saba, Narendra N. Khanna, Mannudeep K. Kalra, Mostafa M. Fouda, Jasjit S. Suri

**Affiliations:** 1 School of Biomedical Engineering, Indian Institute of Technology (BHU), Varanasi 221005, India; 2 Department of Radiology, Azienda Ospedaliero Universitaria (A.O.U.), 100015 Cagliari, Italy; 3 Department of Cardiology, Indraprastha APOLLO Hospitals, New Delhi 110020, India; 4 Department of Radiology, Massachusetts General Hospital, Boston, MA 02115, USA; 5 Department of ECE, Idaho State University, Pocatello, ID 83209, USA; 6 Stroke Diagnostic and Monitoring Division, AtheroPointTM, Roseville, CA 95661, USA; 7 Knowledge Engineering Center, Global Biomedical Technologies, Inc., Roseville, CA 95661, USA

**Keywords:** COVID-19, chest X-ray, classification, regulatory, precision, error rate, deep learning, segmentation, UNet, Xception

## Abstract

Background and Motivation: COVID-19 has resulted in a massive loss of life during the last two years. The current imaging-based diagnostic methods for COVID-19 detection in multiclass pneumonia-type chest X-rays are not so successful in clinical practice due to high error rates. Our hypothesis states that if we can have a segmentation-based classification error rate <5%, typically adopted for 510 (K) regulatory purposes, the diagnostic system can be adapted in clinical settings. Method: This study proposes 16 types of segmentation-based classification deep learning-based systems for automatic, rapid, and precise detection of COVID-19. The two deep learning-based segmentation networks, namely UNet and UNet+, along with eight classification models, namely VGG16, VGG19, Xception, InceptionV3, Densenet201, NASNetMobile, Resnet50, and MobileNet, were applied to select the best-suited combination of networks. Using the cross-entropy loss function, the system performance was evaluated by Dice, Jaccard, area-under-the-curve (AUC), and receiver operating characteristics (ROC) and validated using Grad-CAM in explainable AI framework. Results: The best performing segmentation model was UNet, which exhibited the accuracy, loss, Dice, Jaccard, and AUC of 96.35%, 0.15%, 94.88%, 90.38%, and 0.99 (*p*-value <0.0001), respectively. The best performing segmentation-based classification model was UNet+Xception, which exhibited the accuracy, precision, recall, F1-score, and AUC of 97.45%, 97.46%, 97.45%, 97.43%, and 0.998 (*p*-value <0.0001), respectively. Our system outperformed existing methods for segmentation-based classification models. The mean improvement of the UNet+Xception system over all the remaining studies was 8.27%. Conclusion: The segmentation-based classification is a viable option as the hypothesis (error rate <5%) holds true and is thus adaptable in clinical practice.

## 1. Introduction

COVID-19 is a highly infectious disease caused by the severe acute respiratory syndrome coronavirus 2 (SARS-CoV-2). After the first case was identified in December 2019 in Wuhan, China [1], the virus spread rapidly worldwide, leading to the COVID-19 pandemic in March 2020 [2]. Out of coronavirus-infected people, 81% develop minor to moderate symptoms such as mild pneumonia, 14% develop severe symptoms such as dyspnea or hypoxia, and 5% develop acute symptoms such as shock, respiratory failure, or multiorgan dysfunction [3,4,5,6] such as myocardial injury [7] or vascular injury [8] that may ultimately lead to the death. The standard diagnostic method for COVID-19 is the detection of the virus nucleic acid in a nasopharyngeal sample by RT-PCR (real-time reverse transcription-polymerase chain reaction), RT-LAMP (reverse transcription loop-mediated isothermal amplification), or TMA (transcription-mediated amplification). However, all these procedures are laborious, rigorous, complicated, time-consuming, and costly, with a significantly high error rate [9].

Medical imaging techniques are one of the most fruitful options for detecting infections, diseases, or lesions present in the internal sensitive organs or other body parts [10]. X-rays [11], CT scans [12], MRI [13], and ultrasounds [14] are most commonly adopted for imaging modalities. Chest X-rays and CT scans are also being used to detect COVID-19 infection, the severity or stage of the infection, and the level of lung involvement or impairment after the infection. Chest X-ray has an advantage over CT in having low radiation dose, being cost-effective, easy availability, and fast results [15].

The addition of artificial intelligence, especially deep learning techniques, into medical imaging is one of the miracles in the 21st century for medical diagnosis [16,17,18,19]. Employing deep learning techniques in medical imaging has significantly contributed to precise accuracy, rapid detection, and lowering medical burden, workforce, and human error [20]. For several disease conditions, such as tumor detection [21], breast cancer and lung cancer prognosis, AI and computer-aided diagnosis (CAD) systems have already been approved and accepted by the medical community [22,23].

For COVID-19 detection in chest X-rays and CT, the AI and deep learning methods have shown tremendous successful classification accuracies [24,25,26,27]. However, most of them conducted classification without lung segmentation or executed only two or three-class (pneumonia types) classification [28,29]. Some authors reported work on the segmentation-based classification model (since they remove unwanted regions of interest), however, high accuracy to qualify for a clinical setup in a multiclass framework was still missing [30,31,32,33]. As a result, these systems cannot be adapted for clinical practice since they cannot meet the regulatory requirements of an error rate <5%. Our hypothesis states that if we can design a segmentation-based classification system having an error rate < 5%, typically adopted for 510 (K) regulatory purposes, the diagnostic system can be adapted in clinical settings [34,35,36].

Recently, the UNet system has shown a powerful solution for segmentation in several applications [37,38,39]. We also utilized a large number of CXR scans so that our system could be more generalized, stable and robust. Further, classification models such as VGG16, VGG19, and Xception have shown their ability to classify well [40,41]. We have applied 16 deep learning-based segmentation-based classifications comprising of two deep learning-based segmentation networks, namely: UNet and UNet+, along with eight classification models, namely VGG16, VGG19, Xception, InceptionV3, Densenet201, NASNetMobile, Resnet50, and MobileNet models for achieving the most precise results. Additionally, we used an explainable AI method based on the Grad-CAM heatmap to detect and manifest the lesion present in the X-ray scans.

## 2. Methodology

**Figure 1:**
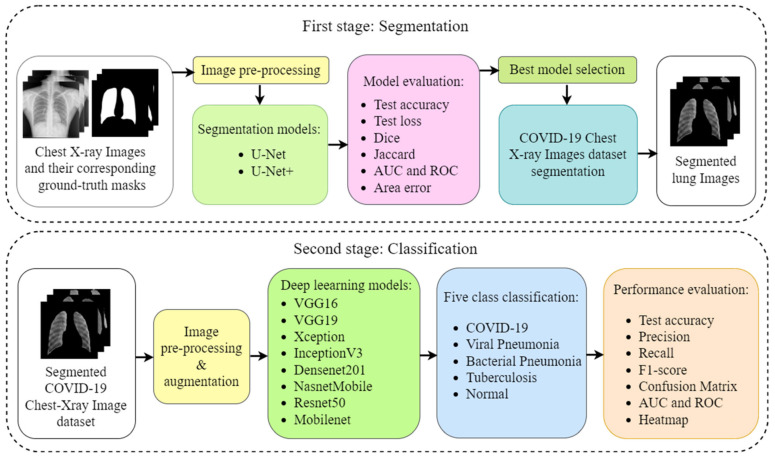
The step-wise overall schematic diagram for the proposed method. (ROC: receiver operating characteristics, AUC: area under curve, VGG: Visual geometry group).

Figure 1 shows the overall methodology we opted for the experiment. The overall experiment was accomplished in two phases. The first phase deals with segmentation, and the second phase with classification. Four different datasets were utilized in the experiment. The first dataset with X-ray images and their corresponding masks was used to train the two segmentation models, namely, UNet and UNet+. The other three datasets were utilized for classification purposes. The best segmentation model was selected and applied to the eight kinds of classification models, namely: VGG16, VGG19, Xception, InceptionV3, Densenet201, NASNetMobile, Resnet50, and MobileNet, for classification into five classes: COVID-19, viral pneumonia, bacterial pneumonia, tuberculosis, and normal (controls). The performance of all the classification models was evaluated using several parameters, including the accuracy, area-under-the-curve (AUC), confusion matrix, and heatmap visualization of the images under explainable AI framework. For our COVID-19 detection system, the best combination of networks for segmentation and classification was finally chosen.

### 2.1. Segmentation

#### 2.1.1. Data Collection and Patient Demographics

**Figure 2:**
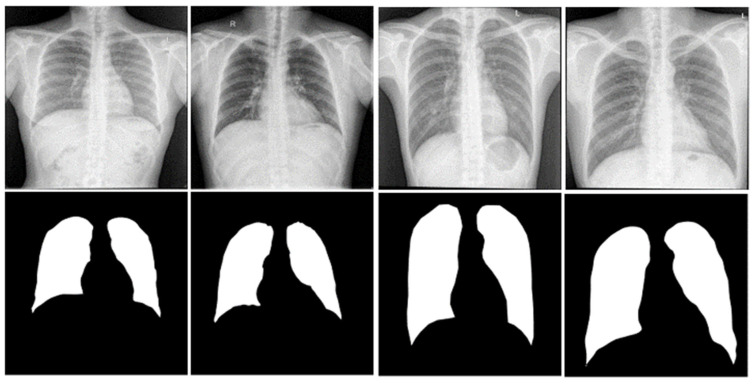
Sample CXR images (top row) and their ground truth masks (bottom row).

In this work, the Kaggle dataset named: ‘Chest Xray Masks and Labels’ was used to train the segmentation models [42]. The dataset contains 704 chest X-ray images and their corresponding ground truth masks. A team of expert radiologists annotated each mask. The data source is the National Library of Medicine, NIH, USA, and Shenzhen No. 3 People’s Hospital, GMC, Shenzhen, China. The dataset contains 360 normal chest X-rays and 344 infected chest X-ray images. Figure 2 shows sample chest X-ray images and their corresponding masks.

#### 2.1.2. Segmentation Model Selection

The UNet network has been shown to be powerful for lung region segmentation in X-ray scans [43,44]. The model is ideal due to its ability to extract the grayscale features in supervised-based segmentation. The power of contextual and semantic features in low-lying layers and high-lying layers allows UNet-based architecture to extract the feature in segmentation paradigm. The concatenation phase via skip connection allows for recovery of the best features from the encoders. The upsampling in the decoder phase is equally powerful for reconstruction of the image size while retaining the features. The UNet+ model has some intermediate encoder stages between compression and expansion. The intermediate up-sampling units with varying depths in the UNet+ model have overcome the limitation of optimal depth in the UNet encoder-decoder network. These days, advanced hybrid models are in the pipeline which uses UNet in combination with other networks such as SegNet-UNet [38], ResNet-UNet, VGG-UNet [39], SegNet-UNet+ [45]. Some attention UNet models are also very popular in recent days that use UNet as a backbone for the segmentation of medical images [46,47]. Based on their popularity, compatibility, reliability, and results we selected both UNet and UNet+ networks for the segmentation of the chest X-ray images.

#### 2.1.3. The Architecture of Segmentation Networks

Two deep neural network models, namely UNet and UNet+, were applied for our first experimental phase, i.e., segmentation of chest X-ray images.

**Figure 3:**
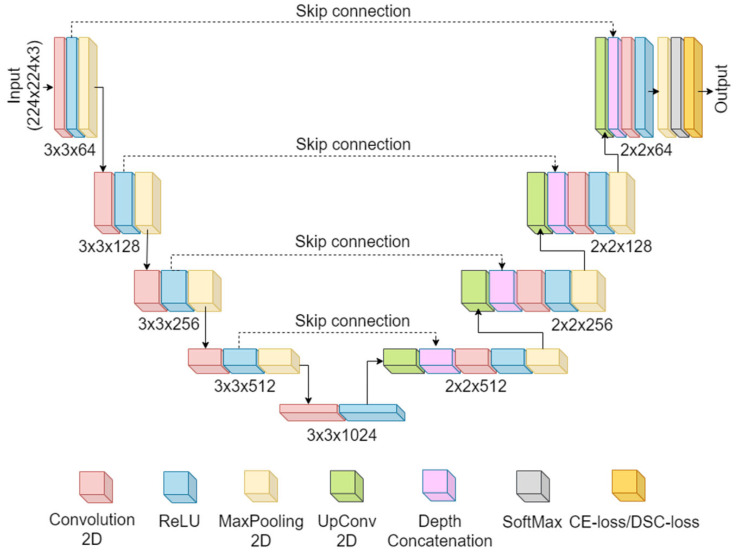
UNet architecture.

UNet architecture: UNet is the most popular convolutional neural network for segmentation. It was proposed by Ronneberger et al. [48]. The network applies the idea of deconvolution, which was introduced by Zeiler et al. [49]. Figure 3 represents the UNet architecture. It consists of a blend of encoder-decoder stages. The encoder encompasses a combination of convolutional layers followed by the ReLU and Maxpooling. The encoder has a 3 × 3 convolution with a MaxPooling that downsamples the images to the next stage and finally to the bridge network. The bridge network is present at the bottom of the U-shaped network that connects the encoder with the decoder. The bridge network has 3 × 3 × 1024 filters and a ReLU layer. Next to the bridge stage, the decoder functions by up-sampling the images. The decoder comprises up-convolution, convolution, ReLU, and MaxPooling layers. Each decoder stage has 2 × 2 convolutional filters. The spatial features from the encoder stage are delivered (transferred) to the corresponding decoder stage through a skip connection [50]. After the fourth decoder stage, the ADAM optimizer contributes to reducing the loss. Finally, an efficient classifier, the Softmax, classifies the up-sampled features into two classes: the lung area and the background.

**Figure 4:**
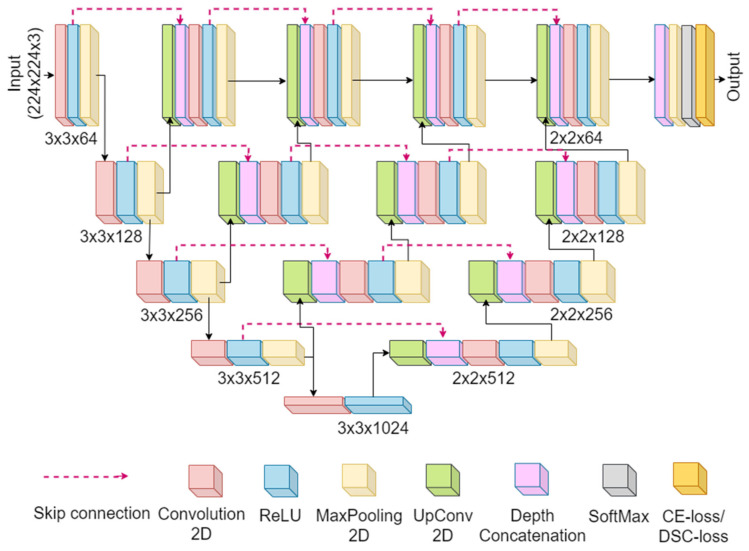
UNet+ architecture.

UNet+ architecture: The UNet+ is a modified version of the UNet network. Figure 4 represents the UNet+ model. The UNet+ model differs from the original UNet by having a few intermediate encoder stages between compression and expansion. The first intermediate stage has three encoder stages, the second has two, and the third has one intermediate stage. The several intermediate up-sampling units with varying depths in the UNet+ model have overcome the limitation of optimal depth in the UNet encoder-decoder network. All intermediate up-sampling units are connected to the decoder stage with the exact resolution by reformed skip connections. Finally, after the fourth decoder stage, similar to the UNet, the ADAM optimizer reduces the loss, and the Softmax classifies the up-sampled features into two classes: the lung area and the background.

#### 2.1.4. Experimental Protocols

Cross-validation: A total of 704 CXR images and their 704 corresponding masks were used for the segmentation experiment. The K5 data partitioning method was implemented. The 5-fold cross-validation is the most popular protocol, where 80% of images are used for training and 20% for testing the model [51,52]. The cross-validation technique is most useful if the number of available images for training and testing is low. In the small size, dataset cross-validation enables the utilization of each image for training and each image for testing at least once. This prevents a high error in the results; therefore, the most reliable results could be achieved. Our segmentation dataset has 704 images that are also relatively low in numbers, which is why using cross-validation enables our results to be more reliable. The 5-fold cross-validation was performed utilizing 80% of images for the training part (60%, i.e., 408 images for training, and 20%, i.e., 148 images for validation). The 20%, i.e., 148 images were utilized for testing the model in each fold. After each fold’s training and validation, testing was performed on 148 new images that were not used in training or validation. The average test results for each fold were calculated to obtain the performance analysis, including the test accuracy and loss. In addition, the mask was generated for images of the test set using each model developed by training on each fold’s images. Next, all of the predicted masks from each fold’s test images were compared with their ground truth masks to see how well they worked. This was done by generating the Dice, Jaccard, area error, Bland–Altman plot, coefficient of correlation, and receiver operating characteristics (ROC).

#### 2.1.5. Training and Loss Function

Both the UNet and UNet+ models were trained for 100 epochs with a learning rate of 0.001, a dropout rate of 0.25, and a batch size of 4 images. The loss function used for training the model was the CE (Cross-Entropy) loss function. Cross-Entropy is the method for computing the error between the binary output stage of the segmentation process and the ground truth image [53]. The output stage is the stage when the forward propagation is over, and backpropagation is about to begin. The cross-entropy is mathematically given as a function of (a) log() function of the predicted label and (b) the gold standard [54]. The Cross-Entropy loss function is denoted by L_ce_ and mathematically represented as [37]:

(1) $$L_{\mathrm{ce}}=\left[\left(y_{i}\times {\mathrm{loga}}_{i}\right)+\left(1-y_{i}\right)\times \log(1-a_{i}\right)]$$

where, y_i_ is the input GT label 1, (1 − y_i_) is GT label 0, and a_i_ represents the Softmax classifier probability.

The entire experiment was conducted using Python 3.8. For training the network, we employed a workstation with an 8 GB NVIDIA Quadro P4000 Graphics Processing Unit (GPU). The system had an Intel Core i7 8th Generation processor and 16 GB of RAM.

#### 2.1.6. Performance Evaluation Metrics

The performance of each network for image segmentation was evaluated on test data after the training and validation process. The following different matrices were utilized for the performance evaluation naming: accuracy, loss, Jaccard index, Dice coefficient, area error, and AUC. The mathematical representations for the matrices are given in the equation below [37,55,56]:

(2) $$\mathrm{Accuracy}=\frac{\mathrm{TP}+\mathrm{TN}}{\;\left(\mathrm{TP}\;+\;\mathrm{FN}\right)+\left(\mathrm{FP}\;+\;\mathrm{TN}\right)}$$

(3) $$\mathrm{Jaccard}\;\mathrm{index}\;=\frac{\mathrm{TP}}{\;\left(\mathrm{TP}\;+\;\mathrm{FN}+\mathrm{FP}\right)}$$

(4) $$\mathrm{Dice}\;\mathrm{Coefficient}=\frac{\left(2*\mathrm{TP}\right)}{\;\left(2*\mathrm{TP}\;+\;\mathrm{FN}+\mathrm{FP}\right)}$$

where TP: True Positive, TN: True Negative, FP: False Positive, FN: False Negative, TP represents the number of COVID-19 samples correctly classified as COVID-19, while FN represents the number of COVID-19 samples wrongly classified to other class. Similarly, TN represents the number of other class samples correctly classified to respective class while FP represents the number of other class samples wrongly classified as COVID-19.

### 2.2. Classification

#### 2.2.1. Data Collection and Patient Demographics

For the classification phase of experiment, a total of 12,926 chest X-ray images were used. The images were taken from three different publicly available data sources, which are: “COVID-19 Radiography Database” [57], “Tuberculosis (TB) Chest X-ray Database” [58], and “Chest X-Ray Images (Pneumonia) [59]”. The “COVID-19 Radiography database” contains 3616 COVID-19, 1345 viral pneumonia, and 10,192 normal images. The dataset was created by a group of researchers and doctors from Bangladesh, Pakistan, and Malaysia [60,61]. From the dataset, we have taken 3611 COVID-19, 1345 viral pneumonia, and 4490 normal images for the experiment. The “Chest X-ray images (Pneumonia)” dataset contains 5863 images, with 2780 bacterial pneumonia images. The chest radiographs were taken from the Guangzhou Women and Children’s Medical Center, Guangzhou [62,63]. From the dataset, we have taken all the 2780 bacterial pneumonia radiographs for the experiment. Next, the “Tuberculosis Chest X-ray Database” contains 700 tuberculosis chest X-rays. The database was created by the collaboration of several groups of researchers and doctors [56]. We have utilized all the 700 radiographs of tuberculosis for our experiment.

Finally, 12,926 total CXR images having COVID-19, viral pneumonia (VP), bacterial pneumonia (BP), Tuberculosis (TB), and normal images of 3611, 1345, 2780, 700, and 4490, respectively, were utilized for our classification experiment. All the CXR images were segmented before the classification. For training of the classification model, 80%, i.e., 10,338 total images having COVID-19, VP, BP, TB, and normal images of 2887, 1075, 2224, 560, and 3592, respectively, were utilized. Next, for validation of the model, 10%, i.e., 1294 randomly selected images, of which COVID-19, VP, BP, TB, and normal images of 362, 135, 278, 70, and 449, respectively, were utilized. Finally, for testing the model, 10%, i.e., 1294 randomly selected images that were not involved in training nor in validation and included COVID-19, VP, BP, TB, and normal images of 362, 135, 278, 70, and 449, respectively, were utilized.

#### 2.2.2. Classification Model Selection

Our main focus was towards the design of the clinical-based system, thus the key aspect in the choice of the classifier is the performance criteria along with its popularity. We therefore selected eight such classifiers, namely VGG16, VGG19, Xception, Inception V3, DenseNet 201, NasNet Mobile, ResNet 50, and MobileNet. The second reason was an easy interface between the segmentation and the classification pipeline. Our future objective is to move the desktop-based design to cloud-based framework and therefore these high performing classifiers could be the ideal choice for our cloud-based system design [64,65].

#### 2.2.3. The Architecture of Classification Networks

**Figure 5:**
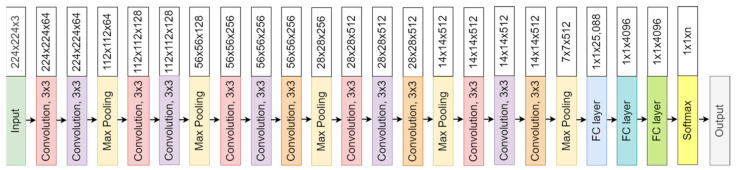
VGG16 architecture.

**Figure 6:**
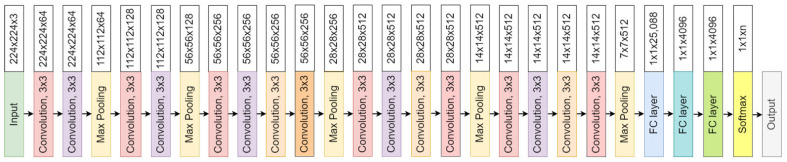
VGG19 architecture.

**Figure 7:**
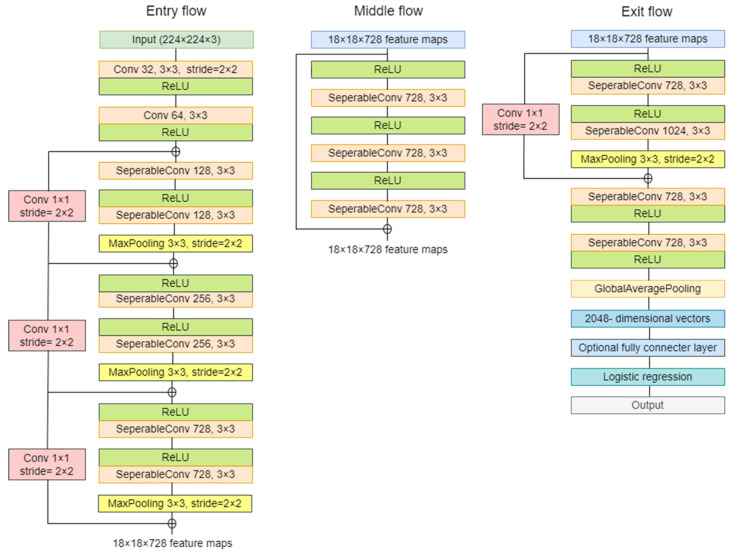
Xception architecture.

**Figure 8:**
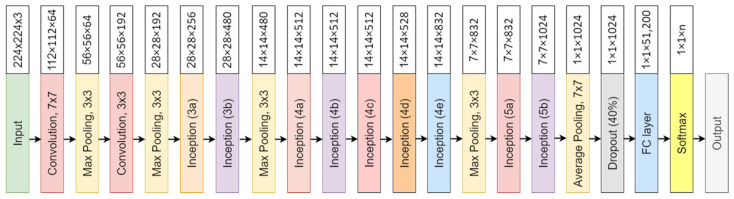
InceptionV3 architecture.

**Figure 9:**
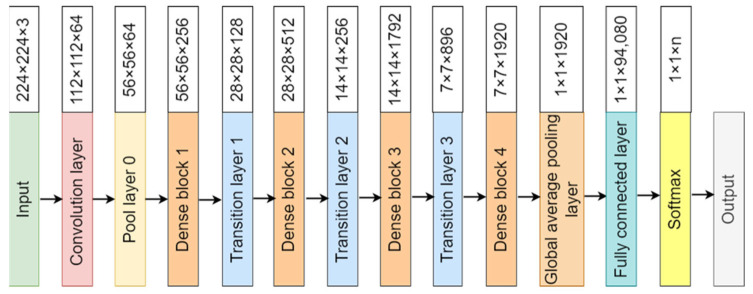
Densenet201 architecture.

**Figure 10:**
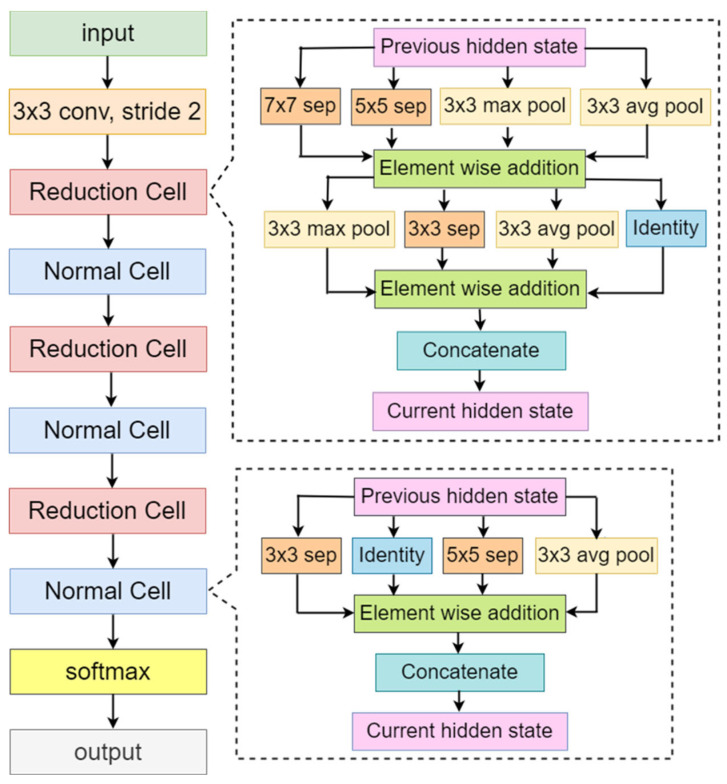
NASNetMobile architecture.

**Figure 11:**
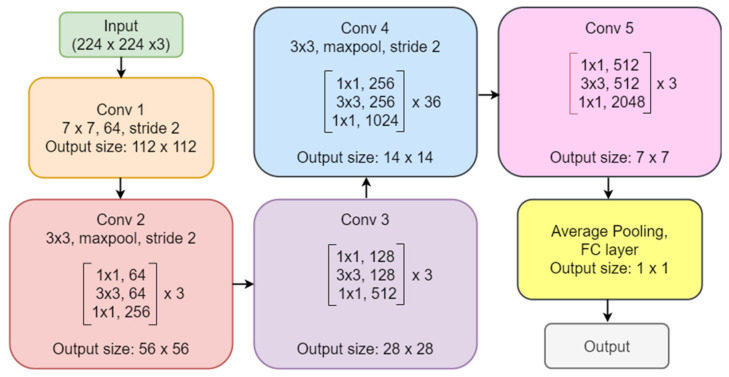
ResNet50 architecture.

**Figure 12:**
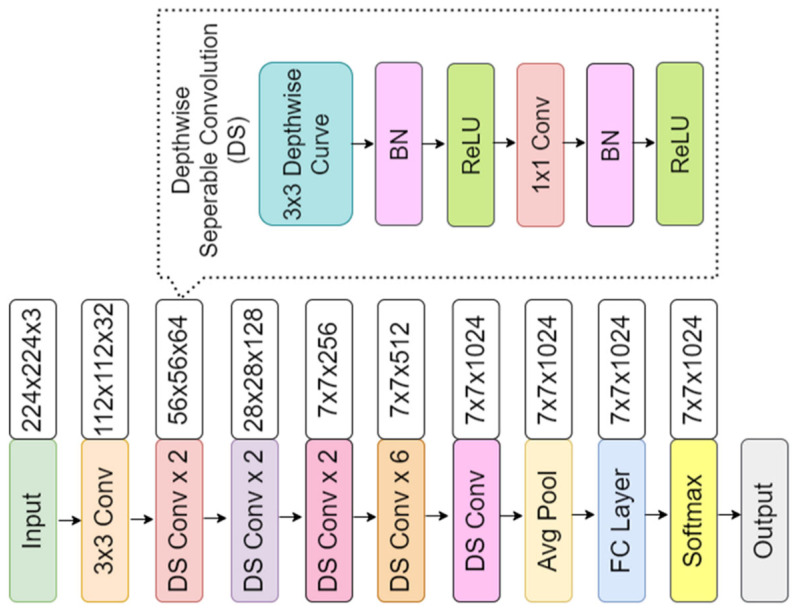
MobileNet architecture.

The convolutional neural network comprises an input, hidden, and output layer. The neural network’s layer works in a feed-forward manner. The intermediary layers are hidden since the activation function and final convolution hide their input and outputs. The hidden layers typically consist of convolution layers followed by activation, pooling, and fully connected layers. The feature maps that are generated by convolution work as input for the next layer. For classification of segmented lung images into five classes, we applied eight highly efficient deep convolutional neural networks namely: VGG16, VGG19, Xception, InceptionV3, Densenet201, NASNetMobile, Resnet50, and MobileNet. The architecture of each neural network is shown in Figure 5, Figure 6, Figure 7, Figure 8, Figure 9, Figure 10, Figure 11 and Figure 12. Each figure describes details about the network’s hidden layers, including convolution layers, their input layer, fully connected (FC)-layers, and output layers.

Figure 5 represents VGG16 architecture. VGG16 is a 16-layer depth model with 13 convolution layers. It has 138 million parameters with a size of 528 MB. It performs with a speed of 4.2 ms per inference step using GPU. Figure 6 represents VGG19 architecture. VGG19 is a slightly larger network than VGG16 and has a depth of 19 layers with 16 convolutional layers. It is 548 MB in size with 143 million parameters. It performs with a speed of 4.4 ms per inference step.

Xception is an 81-layer depth model represented in Figure 7. It has 22.9 million parameters with a size of 88 MB. It performs with a speed of 8.1 ms per inference step. Figure 8 represents InceptionV3 architecture. It is a 189 layers depth model. InceptionV3 is a 92 MB network with 23 million parameters. Its speed is 6.9ms per inference step.

Figure 9 represents DenseNet201 architecture. DenseNet201 is the highest in-depth, with 402 layers. However, it is smaller in size in comparison to others, having 8 million parameters with 33 MB of size. It provides a speed of 5.4 ms per inference step. Figure 10 represents NASNetMobile architecture.

NASNetMobile is the smallest network after MobileNet in all our eight models, even after it has the highest depth after DenseNet201 with 389 layers. It has 5.3 million parameters with 23 MB in size. Its speed is 6.7 ms per inference step. Figure 11 represents ResNet50 architecture. ResNet50 has a depth of 107 layers. It has 25.6 million parameters with 98 MB in size. It provides a speed of 4.6 ms per inference step. MobileNet is the smallest network among all eight models represented in Figure 12. It has a depth of 55 layers with 4.3 million parameters and is 16 MB in size. It is the fastest among all, with a performance of 3.4 ms per inference step. Comparing all eight networks, VGG19 is the largest in size and parameters, Xception is the maximum in-depth, and MobileNet is the fastest network.

#### 2.2.4. Training Parameters

All the models were trained for 150 epochs with a learning rate of 0.001 and a batch size of 8 images. Model checkpoints (save best only) were applied as callbacks. Before the training, all the images were resized to a pixel value of 224 × 224. The loss function used during training was categorical cross-entropy. Categorical cross-entropy is the most popular and important loss function used for multiclass classification tasks [66]. Cross-entropy is an excellent loss function for Classification Problems because it minimizes the distance between two probability distributions—predicted and actual. Ideally, a reliable system is expected to have predicted probabilities close to the original true probability distribution. The categorical cross-entropy makes sure to minimize the difference between all probabilities. The categorical cross-entropy loss function can be defined as the equation below [67]:

(5) $$L_{\mathrm{CCE}}=\frac{1}{N}\;\overset{N}{\underset{i=1}{\sum }}\overset{C}{\underset{c=1}{\sum }}1_{y_{i}\in C_{c}}\log a_{\mathrm{model}}(y_{i}\in C_{c})$$

where, N is the total number of observations (images), C is the number of categories or classes, and $1_{y_{i}\in C_{c}}$ term indicates the ith observation that belongs to the cth category.

The entire experiment was conducted using Python 3.8. For training the network, we employed a workstation with 8 GB NVIDIA Quadro P4000 GPU. The system had an Intel Core i7 8th Generation processor and 16 GB of RAM.

#### 2.2.5. Matrices Used for Result Evaluation

The performance of each network was evaluated on test data after the training and validation process. Five different matrices were utilized for the performance evaluation, namely: accuracy, precision, recall, F1-score, and area under the curve (AUC). The mathematical equations for each matrix are given in the equation below [28,60,68,69]:

(6) $$\mathrm{Accuracy}=\frac{\mathrm{TP}+\mathrm{TN}}{\;\left(\mathrm{TP}\;+\;\mathrm{FN}\right)+\left(\mathrm{FP}\;+\;\mathrm{TN}\right)}$$

(7) $$\mathrm{Precision}=\frac{\mathrm{TP}}{\mathrm{TP}\;+\;\mathrm{FP}}$$

(8) $$\mathrm{Recall}=\frac{\mathrm{TP}}{\mathrm{TP}\;+\;\mathrm{FN}}$$

(9) $$F1-\mathrm{score}=\frac{2\times \;\mathrm{Precision}\;\times \;\mathrm{Recall}}{\;\mathrm{Precision}\;+\;\mathrm{Recall}\;}$$

where TP: True Positive, TN: True Negative, FP: False Positive, and FN: False Negative.

## 3. Results

### 3.1. Segmentation

**Figure 13:**
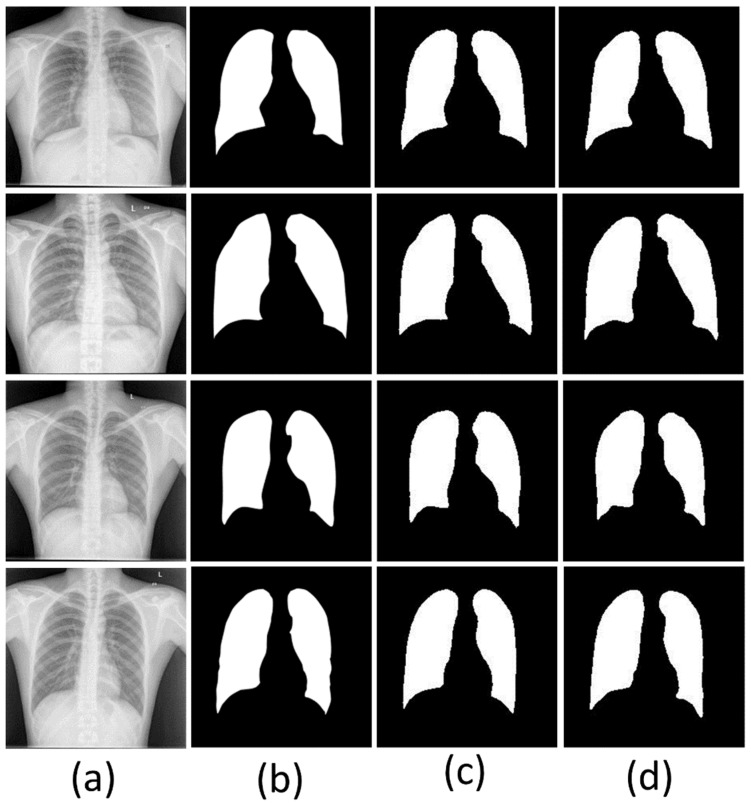
Comparison of results by two segmentation models; (**a**): Original CXR images, (**b**): Ground truth masks, (**c**): Masks generated by UNet model, (**d**): Masks generated by UNet+ model.

Figure 13 shows the masks generated by the UNet and UNet+ models and their comparison to ground truth masks. The comparative performances of both the segmentation models are shown in Table 1. The performance matrices show the average of results generated for test data of each fold from five folds. The UNet model performed with 96.35% accuracy, 0.15% test loss, 94.88% Dice coefficient, 90.38% Jaccard index, 1.48 mm^2^ area error, and 0.99 AUC with *p* < 0.001. The UNet+ model performed with a test accuracy of 96.10%, a test loss of 0.17%, a Dice coefficient of 92.35%, a Jaccard index of 86.07%, an area error of 2.63 mm^2^, and an AUC of 0.98 with *p* < 0.001. The performance of UNet and UNet+ was almost similar in terms of accuracy; UNet performed just 0.25% better than UNet+. However, there was a significant difference in Dice and Jaccard. UNet showed better performance in Dice and Jaccard by 2.53% and 4.31%, respectively, than UNet+. This difference may occur due to the simpler structure of the UNet, including compatibility with the chest X-ray images.

#### 3.1.1. Cumulative Frequency Curves for Dice and Jaccard

**Figure 14:**
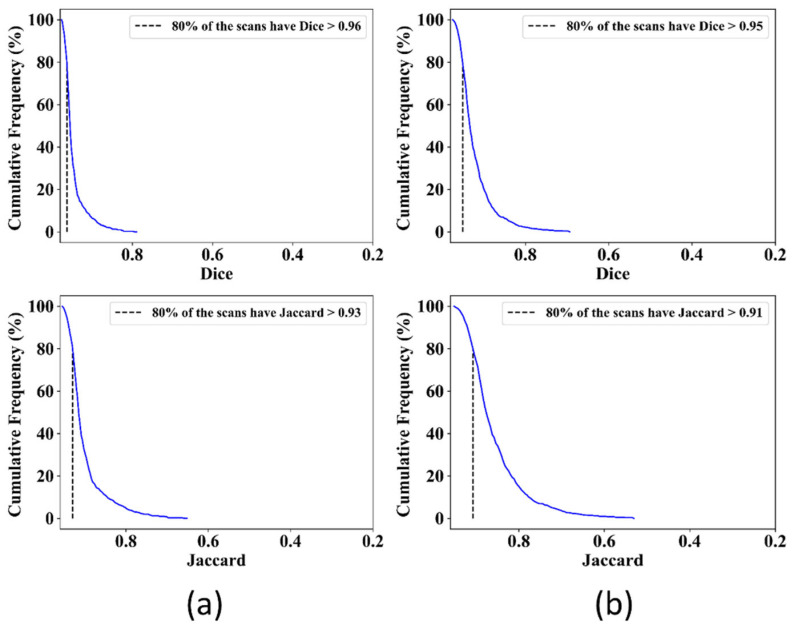
Cumulative Frequency curves showing Dice (top) and Jaccard (bottom) for (**a**): UNet model, (**b**): UNet+ model.

The Dice coefficient or F1-score and the Jaccard index, or intersection over union (IoU), are the most important metrics to evaluate the segmentation. The Dice coefficient is double the area of overlap between AI (predicted mask) and GT (ground truth mask) divided by the total number of pixels in both images. The Jaccard index is the area of overlap between AI and GT divided by the area of union between AI and GT. The Dice and Jaccard are very similar, and both are positively correlated with each other. Figure 14 shows the Cumulative Frequency curves of Dice and Jaccard for both the UNet and UNet+ models.

For the UNet model, 80% of scans had Dice and Jaccard > 0.96 and >0.93, respectively, whereas, for the UNet+ model, 80% of scans had Dice and Jaccard > 0.95 and >0.91, respectively. Thus, the UNet model showed better performance in terms of Dice and Jaccard than the UNet+ model.

#### 3.1.2. Receiver Operating Curve and AUC analysis

**Figure 15:**
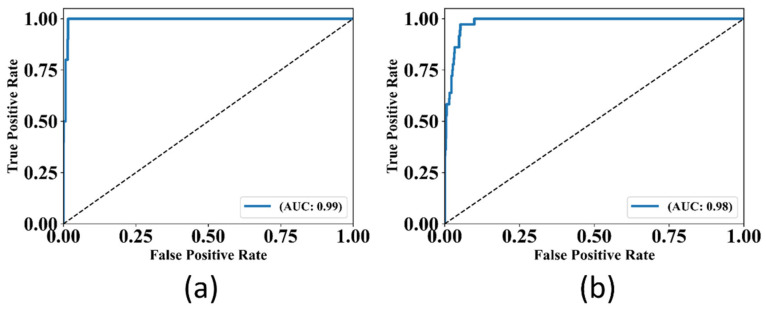
AUC and ROC Curves for (**a**): UNet model, (**b**): UNet+ model (*p* < 0.0001).

The ROC is the graphical plot of sensitivity against the (1-specificity). Higher AUC indicates better performance. Figure 15 shows the ROC and AUC for the UNet and UNet+ models. The AUC performance by the UNet was 0.99, whereas by the UNet+ was 0.98. Thus, the UNet model shows a better ROC curve with a higher AUC value by 1% than the UNet+ model.

#### 3.1.3. Correlation Analysis between AI and GT

**Figure 16:**
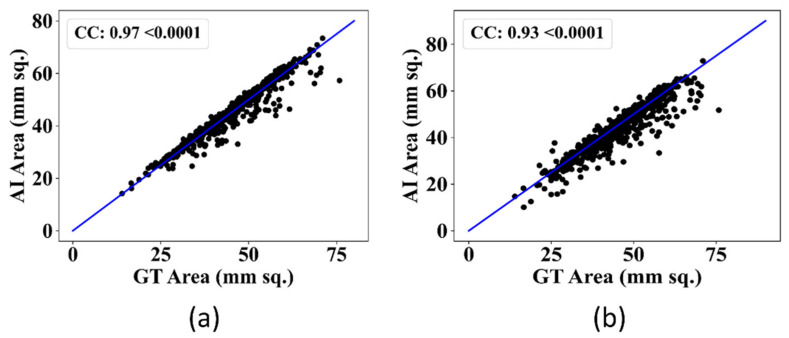
CC for GT and AI-estimated area for (**a**): UNet model, (**b**): UNet+ model.

The regression curve is a prevailing method to find a correlation between two measures. The Correlation coefficient (CC) signifies the relationship between the two measures. The higher CC value denotes a better model performance. Figure 16 shows the CC between AI-estimated and GT area for both models, i.e., UNet and UNet+. The CC value for the UNet model was 0.97, whereas the CC value for the UNet+ model was 0.93. The UNet model showed better performance by 0.04 CC than the UNet+ model.

#### 3.1.4. Bland–Altman Plot for AI and GT Area

**Figure 17:**
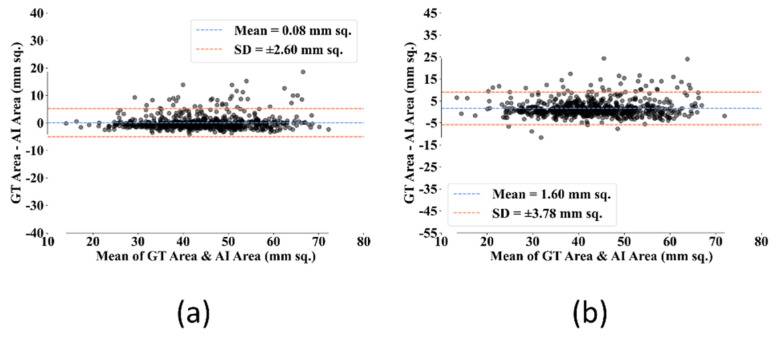
Bland-Altman plots for AI-estimated and GT area for (**a**): UNet model, (**b**): UNet+ model.

The Bland–Altman plot denotes the difference between the AI and GT areas along the *y*-axis and the mean of AI and GT areas along the *x*-axis. The less the mean and SD (standard deviation) values show, the better the performance. Figure 17 shows the Bland–Altman plots for AI-estimated and GT areas for both the UNet and UNet+ models. The mean and SD values for UNet were 0.08 mm^2^ and 2.68 mm^2^, respectively. In contrast, the mean and SD values for the UNet+ model were 1.60 mm^2^ and 3.78 mm^2^, respectively. So, the UNet model performs better than UNet+ by 1.52 mm^2^ and 1.1 mm^2^ in terms of mean and SD, respectively.

#### 3.1.5. Cumulative Distribution Curves for Area Error between AI and GT

**Figure 18:**
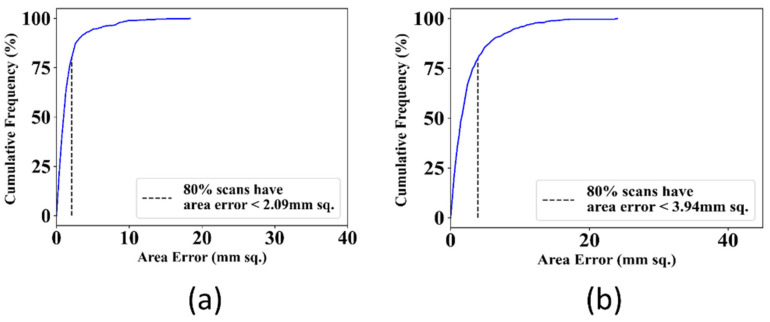
Cumulative distribution curves for area error between GT and AI-estimated masks by (**a**): UNet model, (**b**): UNet+ model.

The area error is one of the other metrics used to determine the model’s performance. The area error is the difference between the area of AI and GT in mm^2^. The area error is calculated by converting the area of predicted and ground truth mask from pixel to mm dimensions and applying a resolution factor of 0.0625 mm to a pixel. A lower error denotes better performance. Figure 18 shows the cumulative distribution curves for area error between GT and AI-estimated masks for both the UNet and UNet+ models. Of the scans, 80% had area error <2.09 mm^2^ for the UNet model whereas 80% scans had area error <3.94 mm^2^ for the UNet+ model. Therefore, the UNet model performed better with less area error of 1.85 mm^2^ than the UNet+ model.

#### 3.1.6. Segmentation of the Classification Dataset

**Figure 19:**
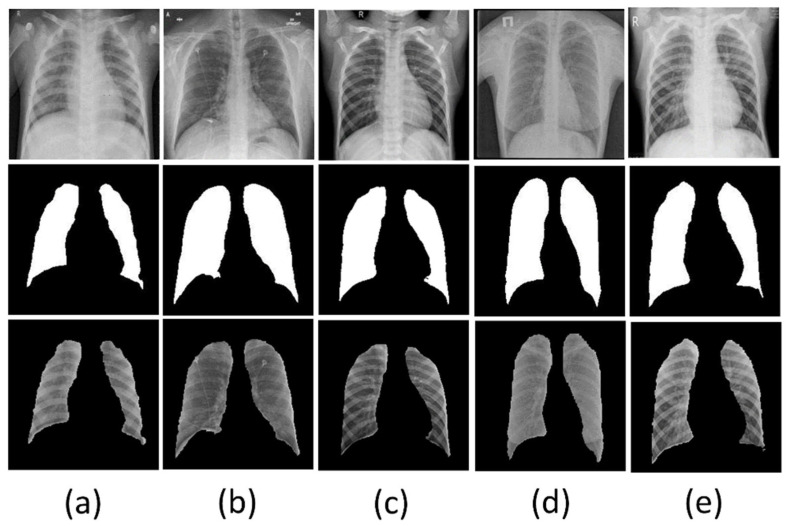
Example of images from classes (**a**): Bacterial Pneumonia, (**b**): COVID-19, (**c**): Normal, (**d**): Tuberculosis, (**e**): Viral Pneumonia; top row: original chest X-ray images, middle row: UNet generated corresponding masks, bottom row: final segmented lung images.

The overall results analysis claims that the UNet model performed better than the UNet+ model in each parameter on our dataset. Therefore, we selected the UNet model for the further segmentation of our classification data. Figure 19 shows the sample of segmented CXR images from the five class classification data by the UNet model.

### 3.2. Classification Results

After the segmentation of classification data, our next goal was to successfully classify and develop a best-suited classification model for the segmented chest X-ray images into five classes with optimal performance. To achieve the goal, we applied eight different highly efficient deep neural networks, namely: VGG16, VGG19, Xception, InceptionV3, Densenet201, NASNetMobile, Resnet50, and MobileNet, for the classification of segmented lung images into five classes: COVID-19, VP, BP, TB, and normal. Table 2 shows the comparison of the performance metrics of all eight CNNs. The Xception model performed best with an accuracy of 97.45% and a weighted average of Precision, Recall, and F1-score of 97.46%, 97.45%, and 97.43%, respectively. Xception is an 81-layer depth model that is highest in terms of depth than other classifiers. Additionally, Xception consists of separable convolution layers that are advantageous over traditional convolutional layers, both in terms of computation cost as well as memory [70]. These features, including the superior accuracy, enable Xception to be the best suited model for our CXR datasets. The performance of MobileNet was the second most efficient, with an accuracy of 93.66% and precision, recall, and F1-score of 93.87%, 93.66%, and 93.60%, respectively. Table 3 shows the performance metrics of each class by the best performing Xception model. The Precision was best for COVID-19 class with 98.88%, whereas the Recall was best for Bacterial Pneumonia class with 100% and F1-score was best for Normal class with 98.55%.

#### 3.2.1. Training and Validation Curve

**Figure 20:**
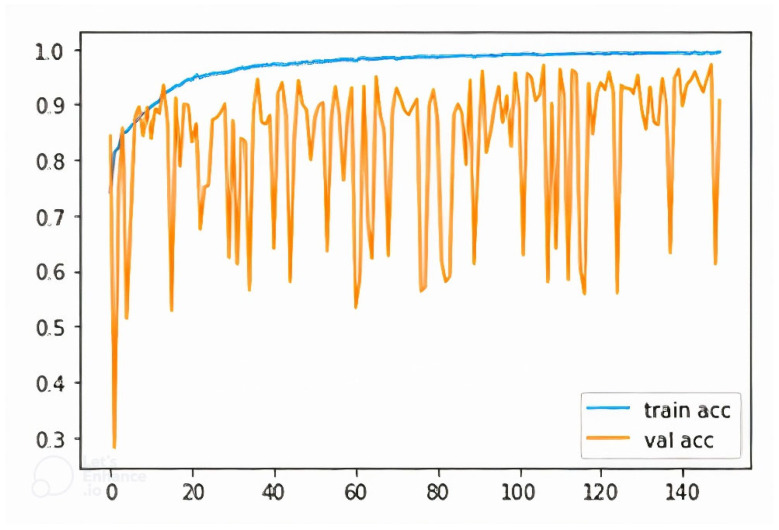
Training and validation accuracy for the best performing Xception model. (train acc: training accuracy, val acc: validation accuracy).

**Figure 21:**
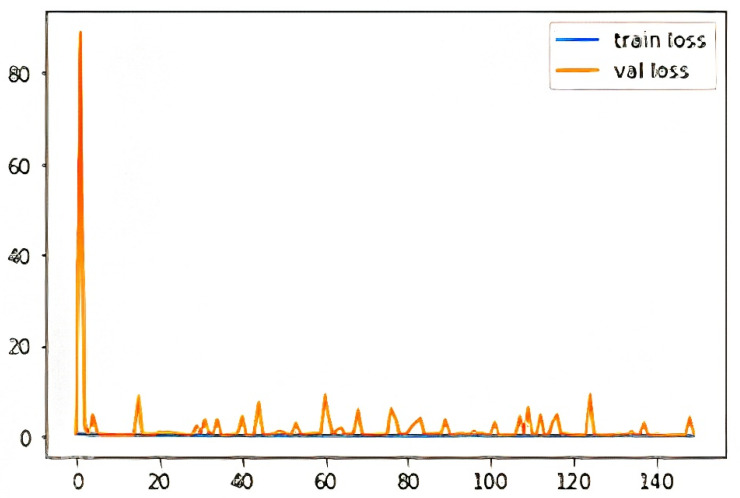
Training and validation loss for the best performing Xception model. (train loss: training loss, val acc: validation loss).

Figure 20 shows the training and validation accuracy for the best performing Xception model. The curve indicates that training and validation accuracy improved with the successive epochs that point towards a good model. Figure 21 shows the training and validation loss curve. The curve indicates that training and validation loss are very stable and reduced with the successive epochs that also supports this as a good model.

#### 3.2.2. Confusion Matrix

**Figure 22:**
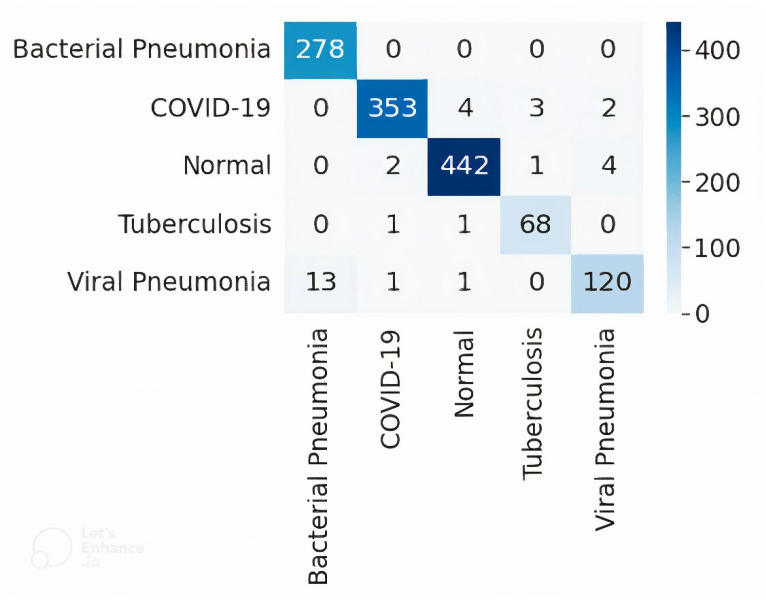
Confusion matrix for five-class classification by the Xception model.

Figure 22 represents the confusion matrix for the test set results by the best performing Xception network. Results reveal that for 362 COVID-19 chest X-ray images, 353 were correctly classified, and nine were misclassified as two to viral pneumonia, three to tuberculosis, and four to normal class. Next, for the viral pneumonia class, out of a total of 135 images, 120 were correctly classified, and 15 were misclassified as one to COVID-19, 13 to bacterial pneumonia, and one to normal class. Further, for the bacterial pneumonia class, all the 278 images were correctly classified. Next, for the tuberculosis class, out of a total of 70 images, 68 were correct, and two were misclassified, with one to COVID-19 and the other to the normal class. Finally, for the normal class, out of 449 images, 442 were correctly predicted, and seven were misclassified with two to COVID-19, four to viral pneumonia, and one to tuberculosis class.

Out of a total of 1294 teat images, just 33 images (~2% only) were misclassified. In addition, out of a total of 362 COVID-19 test images, just nine images (~2% only) were not detected correctly as COVID-19 by our model. This threshold is lower than the regulatory requirement of 5% as per the 510 (K) FDA requirements. The system was designed to meet the regulatory requirement, which is a prerequisite for clinical studies [34,35,36]. The model has passed the regulatory requirement; therefore, it can be used for clinical settings.

#### 3.2.3. Heatmap Visualization: An Explainable AI Model

**Figure 23:**
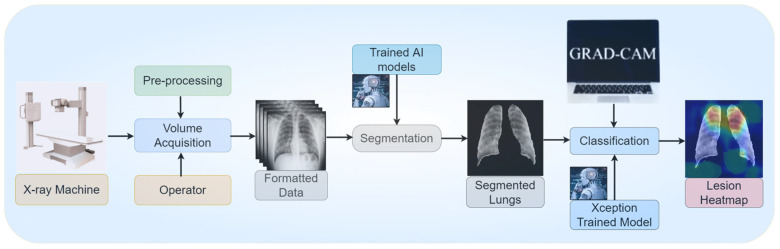
Heatmap generation using the Grad-CAM and the Xception-based classifier.

Lesions have different characteristics such as texture, contrast, intensity variation, density changes, etc. [71]. Figure 23 presents the pipeline for lesion validation using heatmaps, where the input to the segmentation model is the X-ray scans that produce the segmented lungs. This segmented lung goes to the Xception-based classification model for five classes, i.e., COVID-19, viral pneumonia, bacterial pneumonia, tuberculosis, and control [72]. The Gradient-weighted Class Activation Mapping (Grad-CAM) algorithm is applied to produce the lesion heatmap. Grad-CAM builds the coarse localization map using the gradients of the target (COVID-19 in the Xception-based classification model), thereby showing the critical regions in the form of heatmap scans. It uses the final convolution layer to produce the heatmap [64].

**Figure 24:**
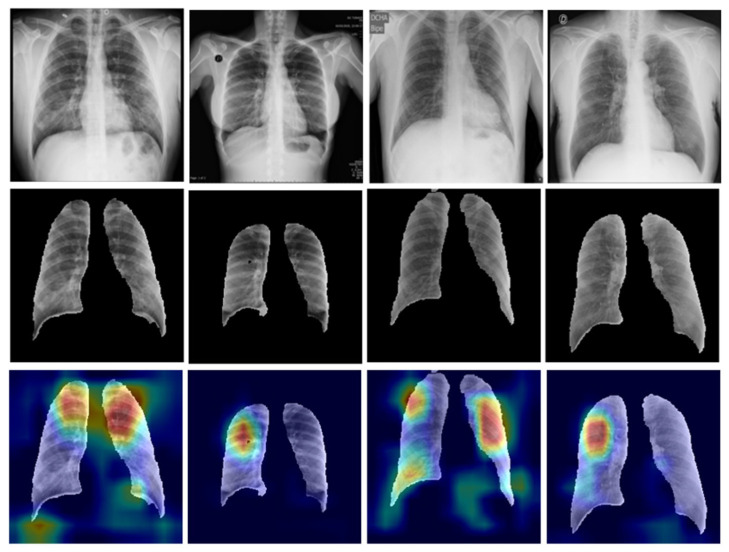
Example of correctly classified COVID-19 chest X-ray images; top row: original COVID-19 infected chest X-ray images, middle row: segmented masks, bottom row: corresponding heat map images.

Heatmaps provide information about from which part of the image the network is learning or distinguishing the images into actual classes. The coronavirus infection starts in the nose or mouth and then infects the throat, trachea, and thereafter the lungs. That is why in most COVID-19 cases and especially during the initial infection stage, the upper part of the lungs is majorly infected. Figure 24 shows the sample images of the COVID-19 class that were correctly classified by the Xception model. The heatmap pattern of the correctly predicted COVID-19 images reveals that the network is distinguishing the images and taking decisions from almost similar parts of the lungs. The model is differentiating the images based mostly on the upper parts of the lungs that are majorly infected or have lesions after the coronavirus infection.

**Figure 25:**
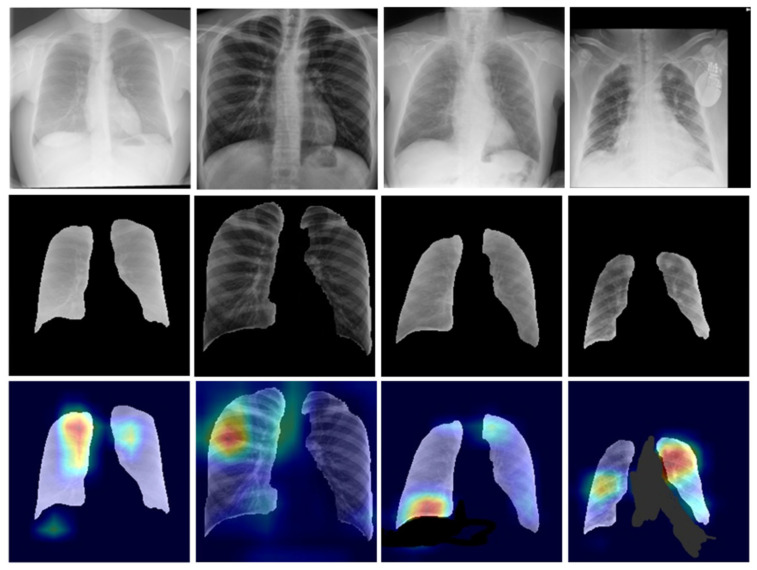
Example of misclassified COVID-19 chest X-ray images; top row: original COVID-19 infected chest X-ray images, middle row: segmented masks, bottom row: corresponding heat map images.

Out of a total of 362 COVID-19 images, nine images (~2%) were misclassified. However, this threshold is lower than the regulatory requirement of 5% as per the 510 (K) FDA requirements. Figure 25 shows some wrongly predicted COVID-19 images and their heatmaps. Sometimes the low contrast or noise present in the images may also be the reason for misclassification.

#### 3.2.4. Performance Evaluation

**Figure 26:**
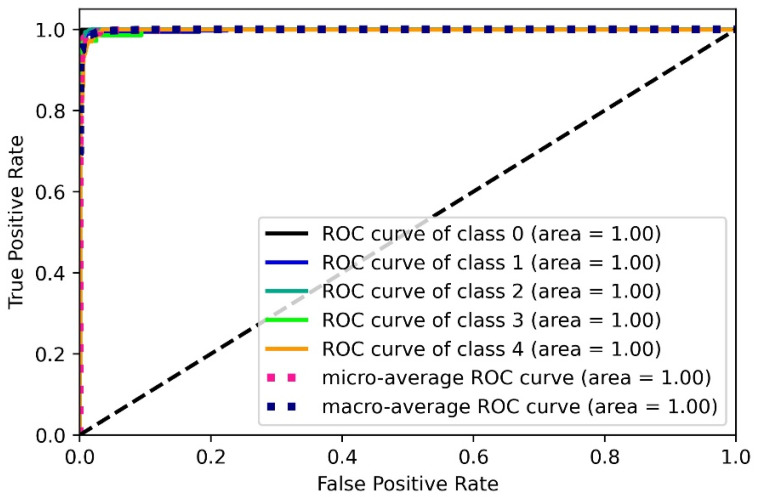
ROC and AUC for the Xception model. (*p* < 0.0001; class 0: BP; class 1: COVID-19; class2: normal; class 3: TB; class 4: VP).

We are able to design a segmentation-based classification model for COVID-19 detection. Our two-stage system has shown excellent performance with precise accuracy in detecting the lesions present in X-ray scans. However, to prove the robustness of the model against all odds, some performance evaluation is always required. Consequently, we obtained the ROC and AUC for the best performing UNet (segmentation) and Xception (classification) models. ROC curves are drawn using inference values and true labels for each class. The ROC and AUC for the UNet model have already been discussed in Section 3.1.2. Figure 26 below shows the ROC and AUC for the Xception model.

## 4. Discussion

### 4.1. Principal Findings

We have developed a two-stage COVID-19 detection system based on the segmentation of CXR images in the first stage and then the classification of the segmented lung in the second stage. Our study consisted of 16 systems (2 segmentation models × 8 classification models). We designed the UNet and UNetPlus-based segmentation models, and this was attempted first time combined with eight types of classification systems in the COVID-19 area. The segmentation step, which consists of UNet and UNet+ blocks, undergoes performance evaluation. The UNet model performed better yielding test accuracy, test loss, Dice, Jaccard, area error, and AUC of 96.35%, 0.15%, 94.88%, 90.38%, 1.48 mm^2^, and 0.99 (*p* < 0.0001), respectively. Next, we applied and tested eight deep neural networks: VGG16, VGG19, Xception, InceptionV3, Densenet201, NASNetMobile, Resnet50, and MobileNet for the classification of the segmented lungs. The Xception model performed the best with accuracy, precision, recall, F1-score, and AUC of 97.45%, 97.46%, 97.45%, 97.43%, and 0.998 (*p* < 0.0001), respectively. Thus, the combination of UNet and Xception is the best-suited model for our system.

Some of the significant outcomes of our system include the following key points: (1) The system is the first of its kind which embeds 16 different configurations, the highest accuracy system beats the previously published in the literature, and the overall improvement was 8.27% compared to the mean of all the available techniques; (2) Our system provided the most robust results based on sensitivity and specificity, the complete pipeline which combines segmentation and classification pairs, uses in cross-validation mode with validation embedded; (3) Further, our system was completely automated, fully scientifically validated and verified; (4) It included an embedded explainable AI component in the segmentation + classification framework; and (5) Finally, the system had an error rate less than 2% which was below 5%, the threshold of the regulatory 510 (K) FDA guidelines for the clinical setting [34,35,36].

### 4.2. Benchmarking for Segmentation Stage

Table 4 shows a comparison of our segmentation model to the existing state-of-the-art segmentation methods. Hooda et al. [73] applied a novel deep CNN on the JSRT CXR dataset and achieved an accuracy of 98.92% with a Jaccard index of 95.88%. Ngo et al. [74] applied a combination of Distance Regularized Level Set and Deep Belief Network to segment the JSRT dataset and achieved an accuracy of 96.5%. Saidy et al. [75] also utilized the JSRT dataset for an encoder-decoder-based segmentation model development and achieved the Dice coefficient of 96% on the test dataset. Mittal et al. [76] utilized the combination of JSRT and Montgomery CXR datasets for an encoder-decoder-based segmentation model and achieved an accuracy of 98.73% and the Jaccard index of 95.10%. Reamarron et al. [77] applied the total variation-based active contour method for the segmentation and used a combination of the JSRT and Montgomery datasets. The model achieved Dice of 89%. Gaal et al. [78] developed a novel segmentation method and applied it to the JSRT dataset. They obtained a Dice coefficient of 97.5%. Munawar et al. [79] utilized three datasets: JSRT, Montgomery, and Shenzhen, for the training of the Generative Adversarial Network and achieved a Dice coefficient of 97.4%. Zhang et al. [80] applied the Dual Encoder Fusion UNet model on a combination of Montgomery and Shenzhen datasets and achieved an accuracy of 98.04% with Dice and an AUC of 96.67% and 0.98, respectively. Teixeira et al. [81] applied the UNet model on the combination of five datasets, namely Cohen, JSRT, Montgomery, Shenzhen, and a private dataset. They achieved a Dice coefficient of 98.2%. Souza et al. [82] applied a combination of AlexNet and ResNet-based CNN segmentation model on the Montgomery dataset and achieved the accuracy, Dice, and Jaccard of 96.67%, 93.56%, and 88.07%, respectively.

In the proposed segmentation method, we utilized a Kaggle dataset naming: Chest X-ray Masks and Labels. The dataset contains 704 CXR images and their corresponding masks. We applied to the UNet network for training. The model performed with a test accuracy, Dice, Jaccard, and AUC of 96.35%, 94.88%, 90.38%, and 0.99 (*p* < 0.000), respectively. Our model performed best in terms of AUC score. In addition, most of the other works utilized JSRT or Montgomery datasets with a deficient number of images, such as 247 and 138, respectively, which may also be reason for some of them have higher accuracy than us. However, we have used a large number of images that make our model more stable and robust.

### 4.3. Benchmarking for Classification Stage

Table 5 compares our classification model to the existing non-segmentation-based classification methods. Nayak et al. [29] applied the ResNet-34 network for the classification of chest X-ray images into COVID-19 and normal classes. They used 203 COVID-19 and 203 normal images taken from GitHub. They achieved an accuracy of 98.33% with an AUC of 0.98. Choudhury et al. [60] utilized the Kaggle dataset for the classification into three classes: COVID-19, VP, and normal by the CheXNet network. They achieved the accuracy of 97.74%. Jain et al. [28] used 490 COVID-19 and 5942 other images for classifying into three classes by the Xception model and achieved an accuracy of 97.97%. Nikolaou et al. [68] used 3616 COVID-19 images for the two and three-class classification of images. They applied the EfficientNetB0 network and achieved an accuracy of 95% for two-class and 93% for three-class classification. Yang et al. [83] applied the VGG16 network to classify into two and three classes. They utilized 3616 COVID-19 and 4845 other images and achieved the accuracy of 98% for two and 97% for three-class classification. Khan et al. [26] applied a novel Coronet model for the classification into three classes and achieved an accuracy of 95%. Hussain et al. [27] used the COVID-R dataset having 500 COVID-19 images, applied a novel CoroDet network for the classification into two, three, and four classes, and achieved the accuracy of 99.1%, 94.2%, and 91.2%, respectively, for each class-type. Aslan et al. [84] applied a hybrid deep learning model having a combination of mAlexNet and BiLSTM (Bidirectional long short term memory) networks on the COVID-19 radiography database having 219 COVID-19 and 2686 other CXR images and achieved an accuracy of 98.7% for the three class classification. Timemy et al. [85] applied the ResNet-50 and Ensemble Subspace Discriminant method for the classification into five classes and achieved the accuracy of 91.6%. Khan et al. [86] applied the EfficientNetB network for the classification into four classes and achieved an accuracy of 96.13%. Our previous work [69] used 3611 COVID-19 and 13,833 other images to classify them into two, three, and five classes. We applied VGG16, NASNetMobile, and DenseNet201 models and achieved an accuracy of 99.84%, 96.63%, and 92.70%, with an AUC of 1.0, 0.97, and 0.92 for two, three, and five-class classifications, respectively.

In the proposed work, we utilized 3611 COVID-19 and 9849 other class images from the Kaggle dataset. We applied the Xception model for the classification after the segmentation by the UNet model. The system performed with accuracy and an AUC of 97.45%, and 0.998, respectively, for the five-class classification. We achieved the highest accuracy and AUC among all previous works for the five-class classification. In addition, we improved the accuracy by 4.75% compared to our previous work. The proposed work also has other several improvements compared to previous work. We have employed segmentation of chest X-ray images before the classification. Further, we have applied the explainable AI-based method and heatmap visualization of the image to detect and manifest the lesion present in the X-ray scans. Additionally, we have applied one new classifier: MobileNet, i.e., the fastest among all involved networks. Finally, as a result, we significantly improved the accuracy, specificity, sensitivity, and AUC compared to our previous work.

### 4.4. Benchmarking for Segmentation-Based Classification

Table 6 below shows the comparison of our system to the existing segmentation-based classification methods. Alom et al. [30] utilized the Kaggle dataset, having 390 COVID-19 images and 234 normal images. They applied a novel NABLA-N network for the segmentation with an accuracy, Dice, and Jaccard of 94.66%, 88.46%, and 86.50%, respectively. Thereafter, the authors applied the Inception Recurrent Residual Neural Network model for the classification of segmented lung images into two classes. They achieved a classification accuracy of 87.26% and an AUC of 0.93. Wehbe et al. [31] utilized a private dataset having 4253 COVID-19 images and 14,778 normal images. They applied an ensemble network for the classification of CXR images after the segmentation. They achieved an accuracy of 83% and an AUC of 0.9 for the two-class classification. Oh et al. [87] utilized 180 COVID-19 and 322 other images taken from Kaggle and GitHub. They applied the DenseNet103 network for the segmentation and achieved the Jaccard of 95.5%. After the segmentation, they applied the ResNet-18 model to classify the segmented lung images into four classes and achieved an accuracy of 88.9%. Teixeira et al. [81] utilized the RYDLS-20-V2 dataset, having 503 COVID-19 and 2175 images from other classes. They applied the UNet model for the segmentation with a Dice coefficient of 98.2%. Following segmentation, they applied Inception V3 for classification into three classes and achieved an accuracy of 88% and AUC of 0.9. Keidar et al. [88] applied the ensemble method for the classification of segmented lung images into two classes. Their model performed with an accuracy of 90.3% and an AUC of 0.96. Fang et al. [55] applied a novel CLseg model for segmentation and achieved the Dice of 94.09%. After the segmentation, they applied a novel SC2Net model for the two-class classification of the COVIDGR 1.0 dataset and achieved an accuracy of 84.23% and an AUC of 0.94. Abdulah et al. [89] applied the Res-CR-Net model for the segmentation with Dice and Jaccard of 98% each. Thereafter, they classified a private dataset into two classes using an ensemble method and achieved an accuracy of 79% and an AUC of 0.85. Bhattacharyya et al. [90] used a GAN segmentation network with a VGG-19 and Random Forest classifier and achieved 96.6% accuracy for the three-class classification. Hertel et al. [91] utilized 4013 COVID-19 with 12,837 other class images. They applied a ResUnet segmentation network with a Dice of 95%. Following segmentation, they applied an ensemble network to classify into two and three classes. They achieved an accuracy of 91% for the two-class and 84% for the three-class with an AUC of 0.95. Aslan et al. [92] applied an ANN based segmentation method on the COVID-19 Radiography database (Kaggle), and combination of DenseNet201 and SVM for the classification into three classes. They achieved an accuracy of 96.29% with an AUC of 0.99. Xu et al. [93] utilized 433 COVID-19 and 6359 other images. They applied ResUNet for the segmentation with a Jaccard of 92.50%. After that, they applied ResNet50 to classify segmented lung images into five classes. They achieved an accuracy of 96.32%.

In our proposed work, we utilized 3611 COVID-19 and 9849 other images taken from Kaggle. We applied the UNet segmentation model and achieved an accuracy, Dice, and Jaccard of 96.35%, 94.88%, and 90.35%, respectively. Thereafter, we applied the Xception model for the classification of the segmented lung into five different classes of pneumonia. We achieved an accuracy of 97.45% and an AUC of 0.998 (*p* < 0.0001). We achieved the highest accuracy and AUC for the segmentation-based classification models among all the existing state-of-the-art methods. This makes our system the most precise, meeting our hypothesis (error rate <5%). Additionally, we have used a large number of images that make our system more stable and robust.

### 4.5. A Special Note on Segmentation-Based Classification of Multiclass Pneumonia

To date, most of the COVID-19 detection systems are based upon the classification of CXR images without segmentation. However, they have shown good accuracy but due to the unwanted region present in the chest X-ray scans, there is the likelihood to have biased or inaccurate results. Segmenting the X-ray images removes the unwanted region and background noise present in the X-ray leaving only the required lung area. Few researchers have worked on the segmentation-based classification model. However, multiclass classification has not been tried, and further, it is not robust in terms of accuracy. Next, note that in previous studies, the number of images used in the experiment for segmentation and even for classification are relatively very low. This may reduce the reliability and robustness of the system. In the proposed work, we tried to fill the gaps by developing a system-based best-suited segmentation-based classification model, keeping regulations in mind. We have used a large number of images for both the segmentation and classification experiments. Additionally, we implemented a classification method that could classify multiple types of pneumonia, including the most common lung infections that generally show similar symptoms and findings in X-rays. If screened using naked eyes by radiologists or doctors, they are very likely to misclassify the different pneumonia types. Even performing multiclass (five-class) classification, our system performed with the highest accuracy compared to any available segmentation+classification model for any class, including two-class. With segmentation, multiclass classification, involvement of the high number of images, and preciseness, our system proves its reliability, robustness, and superiority over other available approaches for medical applications in COVID-19 detection.

### 4.6. Strength, Weakness and Extensions

Our AI-powered system is capable of rapid detection of COVID-19. It takes less than one second to generate the results. Along with the fast detection, our system is more precise than any other available method. The system provides an accuracy of up to 97.45%, which is the maximum among any binary or multiclass segmentation-based classification methods. Additionally, the system designed is highly cost-effective compared to any current diagnostic methods. Our system requires just chest X-ray images that are readily available at a very low cost. The system predicts the disease after segmenting the lung, thus highly accurate meeting regulatory requirements and our hypothesis [94]. Thus, there is less chance of the wrong prediction as most unwanted areas and noises are removed from the X-ray images. Our system can show the infected or lesioned area in the lung by heatmap visualization that may help the radiologists or doctors and ultimately the patients achieve successful treatment. Since our design is AI-based, our system can learn automatically by its own mistakes or by exposure to new images. This constantly continues to enhance the performance of the system. Further, because our system can be easily updated at regular intervals with new sets of images, it can improve the overall performance, especially in diverse data types. For the COVID-19 diagnosis, the setup of our system can be easily created in hospitals or other clinical centers, as it requires just a conventional computer setup and X-ray data sets. Such a system can be adopted for even long-COVID analysis [95]. Such CAD and imaging design can even be extended to the multimodality paradigm. In addition, even a low-skilled person may handle the screening setup without complex training. Our system does not require any sample handling or transportation as in screening using RT-PCR. On the contrary, in our setup, only X-ray images are required that can be transferred in seconds through the internet or other options to any place in the world.

Note that every pilot system design has some kind of challenges. We have noticed that if the resolution of the X-ray images (very low contrast) is beyond the radiologist’s ability to discern pneumonia type, it can affect the AI models. However, this concern can be resolved by denoising and color normalization techniques [96,97]. Furthermore, human error by the X-ray technician may impact the quality of the X-ray image, and ultimately, our AI model’s result might be affected. Sometimes, the variation in X-ray machines and their output quality may affect the results of our system. However, this may be overcome by training on larger data sizes and diverse types of images or by superior de-noising methods [98]. Further, retraining large databases having diverse images, our system would require a high-performing GPU [99] or supercomputer framework [100] that may incorporate higher costs. One significant issue with the AI-based detection system, including ours, is the institutional approval for medical use. Even after many routine developments, the AI-based COVID-19 detection always needs approval as the primary diagnostic method. However, the system may frequently be used as the second opinion choice.

In the extension of the work, we will train our system on more diverse and recent datasets or in a big data framework [101]. More data sets can be collected from different machines and test the performance on the more varied datasets. Superior approaches for training, such as pruning and stochastic imaging to improve the system’s performance and lower the storage [64]. Additionally, we shall use a more advanced GPU and workstations to enhance the output and lower the learning time. Newer methods such as Tree Seed Algorithm (TSA)-optimized Artificial Neural Networks (ANN) can be tried to classify deep architectural features [102]. In another approach, the Bidirectional Long Short-Term Memories (BiLSTM) layer can be used as a hybrid pipeline which combines AlexNet with BiLSTM [84].

## 5. Conclusions

COVID-19 has emerged as one of the predominant challenges to saving human lives in the current circumstances. Several research groups, including medical communities, are trying to find the proper solutions to combat the disease. However, the advancement in artificial intelligence and medical imaging has made hope in lesion detection in medical images. The methods have proved their efficiency in several areas, such as tumor detection, carotid plaque detection, and much more. Numerous research groups are working on AI-based COVID-19 diagnosis systems. However, some gap was still present. In this work, we attempted to fill all the gaps and presented a better two-stage COVID-19 diagnosis system that can fulfill the regulatory requirement of <5% as per the 510 (K) FDA as a prerequisite for clinical settings. We have proposed a segmentation-based multiclass classification system to detect COVID-19 and the other three most common pneumonia, namely viral pneumonia, bacterial pneumonia, and tuberculosis, in chest X-ray scans. We applied two segmentation models: UNet and UNet+, with eight classification networks, namely VGG16, VGG19, Xception, InceptionV3, Densenet201, NASNetMobile, Resnet50, and MobileNet. Finally, we selected the best-performing model combination, UNet for segmentation and Xception for classification. We achieved a classification accuracy of 97.45% with an AUC of 0.998 by the system. Our model outperformed all the existing state-of-art methods in segmentation-based classification models. Our system performed best by the mean improvement of 8.27% over all the remaining studies. Additionally, our system is a completely automated and most robust system yielding the highest sensitivity and specificity. The error rate of the system is just ~2%, which qualifies within the regulatory bounds of less than 5%, a prerequisite for clinical settings. Further, we used heatmaps under the explainable AI paradigm for scientific validation. As our system is more precise, affordable, and accessible than the current diagnostic approaches for COVID-19 and qualifies the regulatory requirement of the FDA, the suggested model may provide an alternative or add to the current diagnostics methods. The system may helpfully aid in rapid and accurate patient diagnosis, reducing the medical workforce and contributing to the wellness of humanity.

## Figures and Tables

**Table 1:** Comparative performance of UNet and UNet+ model.

Model	Test Accuracy (%)	Test Loss (%)	Dice (%)	Jaccard (%)	Area Error (mm^2^)	AUC (*p*-Value)
UNet	96.35	0.15	94.88	90.38	1.48	0.99 (*p* < 0.001)
UNet+	96.10	0.17	92.35	86.07	2.63	0.98 (*p* < 0.001)

**Table 2:** The weighted average of performance metrics by eight different deep learning models for five-class classification of segmented chest X-ray images into COVID-19, VP, BP, TB, and normal.

DL Model	Accuracy (%)	Precision (%)	Recall (%)	F1-Score (%)
VGG16	88.25	87.88	88.25	87.82
VGG19	87.64	87.08	87.64	87.15
Xception	97.45	97.46	97.45	97.43
InceptionV3	90.88	90.89	90.88	90.47
Densenet201	82.07	82.22	82.07	80.90
NASNetMobile	92.97	93.01	92.97	92.78
Resnet50	90.03	90.00	90.03	89.70
MobileNet	93.66	93.87	93.66	93.60

**Table 3:** The performance metrics (precision, recall and F-1 score) for each class for segmented chest X-ray images by the best performing Xception model.

Class	Precision (%)	Recall (%)	F1-Score (%)
Bacterial Pneumonia	95.53	100.00	97.72
COVID	98.88	97.51	98.19
Normal	98.66	98.44	98.55
Tuberculosis	94.44	97.14	95.77
Viral Pneumonia	95.24	88.89	91.95

**Table 4:** Benchmarking table-showing comparison of proposed and existing segmentation models.

Author and Year	Dataset (Chest X-ray)	Technique	Accuracy	Dice	Jaccard	AUC
Hooda et al. (2018) [73]	JSRT	New deep CNN	98.92%	NA	95.88%	NA
Ngo et al. (2015) [74]	JSRT	DRLS (Distance Regularized Level Set) + DBN (Deep Belief Network)	96.5%	NA	NA	NA
Saidy et al. (2018) [75]	JSRT	Encoder-decoder neural network	NA	96%	NA	NA
Mittal et al. (2018) [76]	JSRT+Montgomery	Encoder-decoder neural network	98.73%	NA	95.10%	NA
Reamarron et al. (2020) [77]	JSRT+Montgomery	TVAC (Total Variation-based Active Contour)	NA	89%	NA	NA
Gaal et al. (2020) [78]	JSRT	New deep CNN	NA	97.5%	NA	NA
Munawar et al. (2020) [79]	JSRT+Montgomery+ Shenzhen	GAN (Generative Adversarial Networks)	NA	97.4%	NA	NA
Zhang et al. (2021) [80]	Montgomery+ Shenzhen	DEFUNet (Dual Encoder Fusion UNet)	98.04%	96.67%	NA	0.98
Teixeira et al. (2021) [81]	Cohen v7labs+JSRT+Montgomery+ Shenzhen+Private	UNet	NA	98.2%	NA	NA
Souza et al. (2019) [82]	Montgomery	AlexNet+ResNet based CNN	96.97%	93.56%	88.07%	NA
Proposed	Chest X-Ray Masks and Labels (Kaggle dataset)	UNet	96.35%	94.88%	90.38	0.99

**Table 5:** Benchmarking table-showing comparison of proposed and existing classification (solo) models.

Author and Year	Dataset—Chest X-ray (COVID-19 Images + Other Images)	Technique	Accuracy	AUC
Nayak et al. (2020) [29]	GitHub (203 + 203)	ResNet-34	2 class-98.33%	2 class-0.98
Choudhury et al. (2020) [60]	COVID-19 Radiography database (Kaggle) (423 + 3064)	CheXNet	3 class-97.74%	NA
Jain et al. (2020) [28]	Kaggle (490 + 5942)	Xception	3 class-97.97%	NA
Nikolaou et al. (2021) [68]	COVID-19 Radiography database (Kaggle) (3616 + 11,537)	EfficientNetB0	2 class-95% 3 class-93%	NA
Yang et al. (2021) [83]	COVID-19 Radiography database (Kaggle) (3616 + 4845)	VGG16	2 class-98% 3 class-97%	NA
Khan et al. (2020) [26]	GitHub (284 + 967)	Coronet (novel CNN)	3 class-95%	NA
Hussain et al. (2020) [27]	COVID-R dataset (500 + 1600)	CoroDet (novel CNN)	2 class-99.1% 3 class-94.2% 4class-91.2%	NA
Aslan et al. (2020) [84]	COVID-19 Radiography database (Kaggle) (219 + 2686)	mAlexNet + BiLSTM (Bidirectional long short term memory)	3 class-98.7%	NA
Timemy et al. (2021) [85]	GitHub (435 + 1751)	ResNet-50 + ESD (Ensemble Subspace Discriminant)	5 class- 91.6%	NA
Khan et al. (2022) [86]	COVID-19 Radiography database (Kaggle) (3616 + 17,449)	EfficientNetB	4 class-96.13%	NA
Nillmani et al. (2022) [69]	COVID-19 Radiography database (Kaggle) (3611 + 13,833)	VGG16, NASNetMobile, DenseNet201	2 class-99.84% 3 class-96.63 5 class-92.70	2 class-1.0 3 class-0.97 5 class-0.92
Proposed	COVID-19 Radiography database (Kaggle) (3611 + 9849)	Xception	5 class-97.45%	0.998

**Table 6:** Benchmarking table showing a comparison of proposed and existing segmentation-based classification models.

Author and Year	Segmentation	Dataset—Chest X-ray (COVID-19 Images + Other Images)	Technique	Accuracy	Accuracy Improvement *	AUC
Alom et al. 2020) [30]	NABLA-N network Accuracy—94.66 Dice—88.46 Jaccard—86.50	Kaggle (390 + 234)	Inception Recurrent Residual Neural Network (IRRCNN) model	3 class-87.26%	10.19%	0.93
Wehbe et al. (2021) [31]	NA	Private (4253 + 14,778)	Ensemble CNN	2 class-83%	14.45%	0.9
Oh et al. (2020) [87]	DenseNet103 Jaccard+95.5%	Kaggle + GitHub (180 + 322)	ResNet-18	4 class-88.9%	8.55%	NA
Teixeira et al. (2021) [81]	UNet Dice+98.2%	RYDLS-20-v2 (503 + 2175)	Inception V3	3 class-88% (F1 score)	9.45%	0.9
Keidar et al. (2021) [88]	NA	Private (1289 + 2427)	Ensemble model	2 class-90.3%	7.15%	0.96
Fang et al. (2022) [55]	CLSeg Dice—94.09	COVIDGR 1.0 dataset (426 + 426)	SC2Net (novel CNN)	3 class-84.23%	13.22%	0.94
Abdulah et al. (2021) [89]	Res-CR-Net Dice—98 Jaccard—98	Private (1435 + 3797)	Ensemble CNN	2 class-79%	18.45%	0.85
Bhattacharyya et al. (2021) [90]	GAN network Accuracy—NA	GitHub (342 + 687)	VGG-19 + Random Forest	3 class-96.6%	0.85%	NA
Hertel et al. (2022) [91]	ResUnet Dice—95	COVIDx5 + MIDRC-RICORD-1C + BIMCV dataset (4013 + 12,837)	Ensemble model	2 class-91% 3 class-84%	6.45%	0.95
Aslan et al. (2022) [92]	ANN based segmentation Accuracy—NA	COVID-19 Radiography database (Kaggle) (219 + 2905)	DensenNet201+SVM	3 class-96.29%	1.16%	0.99
Xu et al. (2021) [93]	ResUNet Jaccard—92.50	GitHub (433 + 6359)	ResNet50	5 class-96.32%	1.13%	NA
Proposed	UNet Accuracy—96.35 Dice—94.88 Jaccard—90.38	COVID-19 Radiography database (Kaggle) (3611 + 9849)	Xception	5 class-97.45%	-	0.998

* Accuracy improvement with respect to proposed work.

## Data Availability

The dataset used in this study can be found in references: [42,57,58,59].

## Abbreviations

ADAM	Adaptive Learning Rate Optimization Algorithm
AI	Artificial intelligence
AUC	Area-under-the-curve
BA	Bland–Altman’s Plot
BP	Bacterial pneumonia
CAD	Computer-aided diagnosis
CC	Coefficient of Correlation
CDP	Cumulative Distribution Plot
CE-loss	Cross-Entropy Loss
CNN	Convolution neural network
COV	Coronavirus
CT	Computed tomography
CXR	Chest X-ray
DL	Deep learning
DNN	Deep neural network
ESD	Ensemble subspace discriminant
FC	Fully connected
GPU	Graphics processing unit
IoU	Intersection over union
JPEG	Joint photographic expert group
ML	Machine learning
NasNet	Neural search architecture network
PNG	Portable network graphics
RAM	Random-access memory
ReLU	Rectified linear unit
ResNet	Residual neural network
RF	Resolution Factor
ROC	Receiver operating characteristic
RT-LAMP	Reverse transcription loop-mediated isothermal amplification
RT-PCR	Reverse transcriptase polymerase chain reaction
SARS-CoV-2	Severe acute respiratory syndrome coronavirus 2
TB	Tuberculosis
TMA	Transcription-mediated amplification
VGG	Visual geometry group
VP	Viral pneumonia
WHO	World health organization
2-D	2-dimensional
